# Synergistic protection against hyperoxia-induced lung injury by neutrophils blockade and EC-SOD overexpression

**DOI:** 10.1186/1465-9921-13-58

**Published:** 2012-07-20

**Authors:** Jae H Min, Champa N Codipilly, Sonya Nasim, Edmund J Miller, Mohamed N Ahmed

**Affiliations:** 1 Department of Pediatrics, State University of New York, Downstate Medical Center, Brooklyn, New York, NY, USA; 2 Center for Heart and Lung Research, Feinstein Institute for Medical Research, Manhasset, NY, 11030, USA; 3 Department of Medicine, North Shore University Hospital, Manhasset, NY, 11030, USA; 4 Department of Pediatrics, Cohen Children’s Medical Center at New York, New Hyde Park, NY, USA; 5 Department of Pediatrics, Division of Neonatal-Perinatal medicine, Cohen Children, NS-LIJ Hospitals, 300 Community Dr.Manhasset, Manhasset, NY, 11725, USA

**Keywords:** Extracellular superoxide dismutase, Antileukinate, CXC-chemokine receptor, Hyperoxia, Lung injury

## Abstract

**Background:**

Oxygen may damage the lung directly via generation of reactive oxygen species (ROS) or indirectly via the recruitment of inflammatory cells, especially neutrophils. Overexpression of extracellular superoxide dismutase (EC-SOD) has been shown to protect the lung against hyperoxia in the newborn mouse model. The CXC-chemokine receptor antagonist (Antileukinate) successfully inhibits neutrophil influx into the lung following a variety of pulmonary insults. In this study, we tested the hypothesis that the combined strategy of overexpression of EC-SOD and inhibiting neutrophil influx would reduce the inflammatory response and oxidative stress in the lung after acute hyperoxic exposure more efficiently than either single intervention.

**Methods:**

Neonate transgenic (Tg) (with an extra copy of hEC-SOD) and wild type (WT) were exposed to acute hyperoxia (95% FiO_2_ for 7 days) and compared to matched room air groups. Inflammatory markers (myeloperoxidase, albumin, number of inflammatory cells), oxidative markers (8-isoprostane, ratio of reduced/oxidized glutathione), and histopathology were examined in groups exposed to room air or hyperoxia. During the exposure, some mice received a daily intraperitoneal injection of Antileukinate.

**Results:**

Antileukinate-treated Tg mice had significantly decreased pulmonary inflammation and oxidative stress compared to Antileukinate-treated WT mice (*p* < 0.05) or Antileukinate-non-treated Tg mice (*p* < 0.05).

**Conclusion:**

Combined strategy of EC-SOD and neutrophil influx blockade may have a therapeutic benefit in protecting the lung against acute hyperoxic injury.

## Background

Supplemental oxygen is a common, and life saving, strategy used in neonatal intensive care units [1]. However, exposure to high concentrations of oxygen causes increased oxidative stress [2-4], inflammation [5,6] and damage to lung tissues [2-7]. Persistent exposure to hyperoxia eventually results in irreversible pulmonary toxicity and death [8]. Preterm infants are particularly vulnerable to oxygen toxicity as a consequence of an immature antioxidant system [9,10]. Oxygen may damage lung cells directly via generation of reactive oxygen species (ROS) [11] or indirectly via the action of inflammatory cells and inflammatory mediators [12,13]. These responses, in turn, overwhelm the cellular antioxidant defenses and lead to the accumulation of toxic levels of ROS. Thus, there is a need to develop better treatment strategies to reduce the oxidative burden on the lung, modulate cytokine networks, and prevent recruitment of inflammatory cells that are responsible for the tissue damage.

While supplementing the immature antioxidant system in preterm infants has been investigated, clinical trials of antioxidant vitamin [14] or antioxidant enzyme supplementation [15] have had only modest success or have been ineffective at preventing bronchopulmonary dysplasia (BPD). However, overexpression of manganese superoxide dismutase conferred superior survival in hyperoxia-exposed transgenic (Tg) adult mice [16]. Extracellular superoxide dismutase (EC-SOD) is an antioxidant enzyme that scavenges the potentially harmful superoxide free radical and is highly expressed in the extracellular matrix of lung and vascular tissue in humans and mice. Overexpression or administration of EC-SOD has been shown to protect against a variety of pulmonary insults, including exposure to hyperoxia [17-19], lipopolysaccaride (LPS) [20], and influenza-induced lung injury [21]. EC-SOD is also known to inhibit neutrophil influx in response to hyperoxia [18], LPS [20], bleomycin and bacteria [22].

Cellular injury following oxidative stress provokes an inflammatory response. Pro-inflammatory cytokines such as TNFα and IL-1β are released from the alveolar epithelium, resident macrophages, and other cells following cellular injury [12]. Resident alveolar macrophages orchestrate the initial inflammatory cytokine response and contribute to the initiation of neutrophil influx by secretion of neutrophil chemokines such as IL-8, human growth-related oncogene α (GROα), complement 5a (C5a), platelet activating factor, and platelet derived growth factor. The role of cytokines in coordinating the inflammation, have made them a target for potential therapies for BPD. In addition, administration of neutralizing antibody against neutrophil chemokines coincident with intrapulmonary administration of endotoxin has been found to prevent lung injury in rats, regardless of accompanying high level of proinflammatory cytokines [23]. Therefore, the neutrophil recruitment is noted to be a central effector in the early stages of lung inflammation, further stimulating the accumulation of neutrophil chemokines that provide autocrine signaling, enhancing the oxidative stress by releasing large quantities of extracellular free radicals via NADPH oxidase pathway (respiratory burst), and protease secretion.

Auten et al. demonstrated that neutralizing antibodies against the neutrophil chemokine, cytokine-induced neutrophil chemoattractant-1 (CINC-1) prevented abnormal lung development in newborn rats exposed to 95% O_2_[24]. Furthermore, blocking neutrophil influx also ameliorated nucleic acid damage and DNA oxidation in this model [25]. Liao also described that blocking neutrophil influx by using the CXC-chemokine receptor antagonist (SB265610) protected alveolar development and improved lung function in hyperoxia-exposed newborn rats [26].

Antileukinate, a hexapeptide with acetylated amino-terminus and amidated carboxy-terminus (Ac-RRWWCR-NH,) is a potent inhibitor of CXC receptors 1 and 2 [27]. It specifically inhibits neutrophil chemotaxis and degranulation and has been shown to inhibit lung inflammation in a number of animal models and species [28-34]

While both anti-oxidant and anti-inflammatory strategies have been used separately, each individual approach has only produced a partial protection against hyperoxia. This is the first study to examine, in detail, the protective effects on hyperoxia-induced acute lung injury of combining increased EC-SOD production with inhibition of CXCR 1 and 2 to reduce neutrophil activation.

## Material and methods

### Animals and care

All animal use was approved in advance by the Institutional Animal Care and Use Committee at the Feinstein Institute for Medical Research and was conducted in accordance with guidelines set by the US Animal Welfare Act and National Institutes of Health.

### hEC-SOD transgenic mice

Transgenic B6C3 newborn mice expressing an extra-copy of human EC-SOD were used. The human gene was under the control of the SP-C promoter targeting hEC-SOD overexpression to alveolar type II and nonciliated distal bronchial epithelial cells [18,35].

Tg founders were bred onto the B6C3 strain to obtain several generations of animals and were identified by PCR analysis of tail DNA using the forward primer EC1 (5’-AGACACCTTCCACTCTGAGG-3’) and the reverse primer EC2 (5’-GTTTCGGTACAAATGGAGGC-3’) [18].

### Antileukinate (CXC-chemokine receptor antagonist)

Antileukinate (Ac-RRWWCR-NH) was synthesized by Multiple Peptide Systems (San Diego, CA) and was administered daily with a single intraperitoneal dose of 100 μg/g in 0.1 ml sodium chloride (0.9% w/v) for 7 days beginning at birth. Antileukinate-non-treated mice received sodium chloride alone in a similar fashion. The dose of Antileukinate was based on our previous studies that found similar concentrations to be effective in reducing neutrophil trafficking, and were non-toxic [29,34]. The first dose was given before exposure as soon as litters were born and a total of 7 doses of Antileukinate were given during exposure.

### Exposure to acute normobaric hyperoxia and injection of Antileukinate

Newborn mice (WT and Tg) were randomly assigned into 8 groups (10 pups/group) at birth as follows (Table 1).

**Table 1:** Classification of the eight studied groups based on their genotype, exposure and antileukinate treatment

**Group:**	**1**	**2**	**3**	**4**	**5**	**6**	**7**	**8**
WT	+	-	+	-	+	-	+	-
Tg	-	+	-	+	-	+	-	+
21% (RA)	+	+	-	-	+	+	-	-
95% O_2_ (H)	-	-	+	+	-	-	+	+
Antileukinate	-	-	-	-	+	+	+	+

Two independent experiments were conducted as paired exposures, with one litter receiving 95% O_2_ which was continuously monitored using an O_2_ analyzer, and the other housed in room air. Food and water were available *ad libitum*. Nursing dams were switched between room air and oxygen-exposed litters every 24 hours to prevent maternal O_2_ toxicity. There were no mortalities during the 7 day- hyperoxic exposure.

At 8 days, pups were euthanized with 150 mg/kg pentobarbital sodium *i.p*. Lung tissue and bronchoalveolar lavage (BAL), fluid were obtained. One lung from each mouse was used for biochemical assessments and the contra-lateral lung was used for histopathology.

A. Evaluation of the inflammatory markers:

### BAL cell counts

The trachea was exposed via a midline incision and the lungs were gently lavaged via a tracheal cannula with three aliquots of 0.3 mL Phosphate Buffered Saline (PBS 0.5 M, pH 7.4). The volume of the recovered lavage fluid was recorded, and cell counts were determined using a hemocytometer. Differential counts were performed on cells stained with Wright-Giemsa stain as previously described [36]. Samples with gross hemolysis and with <70% BAL recovery were not analyzed.

### Myeloperoxidase (MPO)

MPO activity was measured spectrophotometrically in lung tissue homogenates by reaction with *o*-dianisidine using a microplate assay [36-38].

### Albumin

The albumin concentration, in BAL fluid, was measured using the assayMax Mouse ELISA kit (Assaypro, St. Charles, MO), following the manufacturer’s instructions.

B. Measurements of oxidative stress:

#### 8-isoprostane

Lung tissue was immediately flash frozen in liquid nitrogen at harvest and stored at −80 °C until analysis to prevent auto-oxidation. Lipid peroxidation is a well-defined mechanism of cellular damage. 8-isoprostane, an indicator of oxidative stress *in vivo* was measured in lung tissue homogenates using Oxiselect 8-iso-prostaglandin F2α ELISA Kit (Cell Biolabs, Inc., San Diego, CA), following the manufacturer’s instructions.

### Ratio of oxidized/reduced glutathione

Reduced and oxidized glutathione was measured in lung tissue homogenates by reaction with DTNB (5, 5’-dithiobis-2-nitrobenzoic acid) using the Glutathione Assay Kit (Calbiochem, Gibbsontown, NJ), following the manufacturer’s instructions.

C. Histological morphometric evaluation:

Harvested lungs were fixed at inflation with 10% phosphate-buffered formalin at 20 cm H_2_O pressure for 30 min. After overnight immersion in fixative, the lungs were embedded in paraffin wax, cut into 5 μm-thick sections, and stained with hematoxylin and eosin. Lungs were examined qualitatively, and a quantitative analysis was performed using light-microscopic morphometry. Five animals from each group were evaluated to determine alveolar surface density and alveolar volume density as estimates of surface area and alveolar number, as described previously [25]. Data from each group are expressed as means ± SE.

### Statistical analysis

Groups were compared using analysis of variance, and a *post hoc* Tukey Kramer test was performed to determine statistical differences. p values less than 0.05 were accepted as significant, assuming an α error = 0.05 and β error = 0.10.

## Results

### The effect of combination of hEC-SOD overexpression and Antileukinate on neutrophil influx into lung after acute hyperoxic exposure

**Figure 1:**
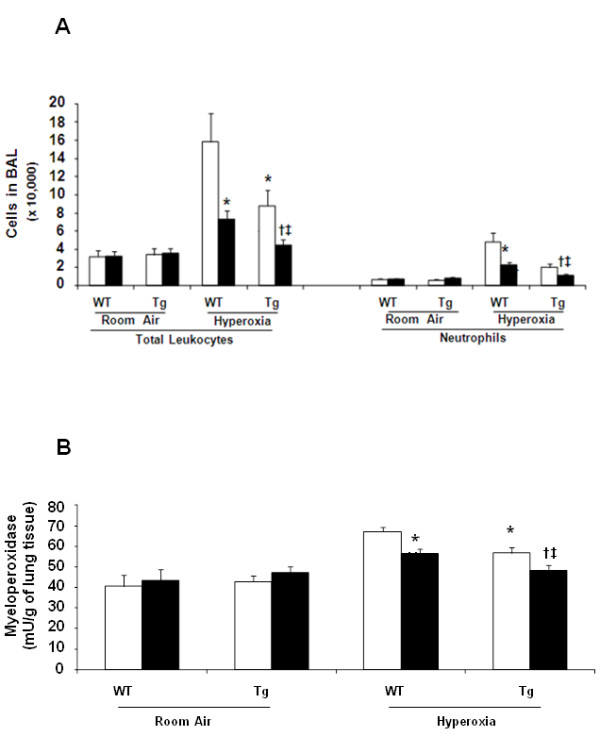
**Leukocyte and Myeloperoxidase in BAL (A) Total number of white blood cells and neutrophils in the BAL fluid and (B) MPO concentration in lung tissue of WT and Tg mice ± Antileukinate (■ treated, □ non-treated) after 7 day-exposure to air or 95% O**_**2**_**.** Antileukinate significantly decreased total white blood cell and neutrophil counts as well as MPO activity in both WT and Tg mice following 95% O_2_ exposure. Tg mice also revealed a significant decrease in total white blood cell and neutrophil count as well as MPO activity compared to WT counterpart groups. Data are mean of ten animals per group ± SEM. * *p* < 0.05 when compared to Antileukinate-non-treated WT/H, ^**†**^*p* < 0.05 when compared to Antileukinate-treated WT/H, ^**‡**^*p* < 0.05 when compared to Antileukinate-non-treated Tg/H.

There was marked elevation in both the total number of white blood cells and neutrophils in the BAL fluid following hyperoxic exposure. In addition, there was increased MPO activity in the lung tissues of both Antileukinate-non-treated WT and Tg mice after acute hyperoxic exposure. In WT mice, Antileukinate significantly reduced neutrophil counts and MPO activity (*p* < 0.05) (Figure 1A&B). Of particular importance, there were significantly less neutrophils and MPO in the BAL of Antileukinate-treated Tg compared to Antileukinate treated WT group after acute hyperoxic exposure (*p* < 0.05).

### The effect of combination of hEC-SOD overexpression and Antileukinate on lung permeability after acute hyperoxic exposure

**Figure 2:**
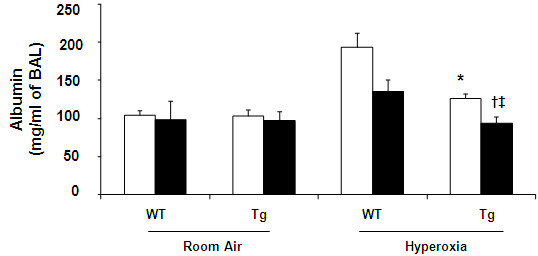
**Albumin concentration in BAL fluid of WT and Tg mice ± Antileukinate (■ treated, □ non-treated) after 7 day-exposure to air or 95% O**_**2**_**.** Antileukinate significantly decreased albumin level in both WT and Tg mice following 95% O_2_ exposure. Tg mice also revealed a significant decrease in albumin level compared to WT counterpart groups. Data are mean of ten animals per group ± SEM. * *p* < 0.05 when compared to Antileukinate-non-treated WT/H, ^**†**^*p* < 0.05 when compared to Antileukinate-treated WT/H, ^**‡**^*p* < 0.05 when compared to Antileukinate-non-treated Tg/H. (RA: room air, H: hyperoxia).

Hyperoxic insult increased the concentration of albumin within the alveolar space, in both WT and Tg mice (Figure 2). In WT mice, Antileukinate significantly reduced the albumin concentration (*p* < 0.05). Antileukinate-treated Tg mice showed significantly decreased albumin levels compared to the Antileukinate-treated WT counterpart group after acute hyperoxic exposure (*p* < 0.05).

### The effect of hEC-SOD overexpression and Antileukinate on oxidative stress

**Figure 3:**
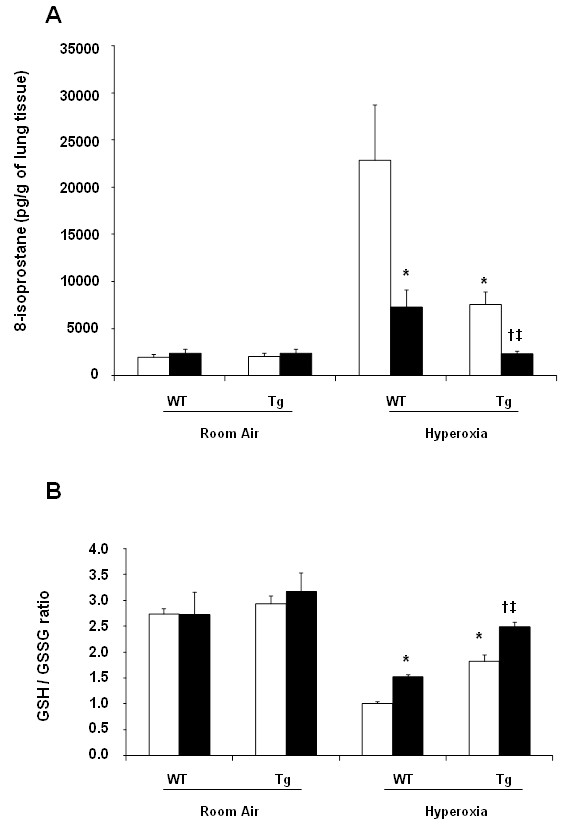
**(A) 8-isoprostane concentration in BAL fluid and (B) The ratio of reduced/oxidized glutathione in lung tissue of WT and Tg mice ± Antileukinate (■ treated, □ non-treated) after 7 day-exposure to air or 95% O**_**2**_**.** Antileukinate significantly decreased 8-isoprostane level and increased the ratio of reduced/oxidized glutathione in both WT and Tg mice following 95% O_2_ exposure. Tg mice also revealed significant decrease in 8-isoprostane level and increase in the ratio of reduced/oxidized glutathione compared to WT counterpart groups. Data are mean of ten animals per group ± SEM. * *p* < 0.05 when compared to Antileukinate-non-treated WT/H, ^**†**^*p* < 0.05 when compared to Antileukinate-treated WT/H, ^**‡**^*p* < 0.05 when compared to Antileukinate-non-treated Tg/H. (RA: room air, H: hyperoxia).

8-isoprostane is an index of oxidative stress in cells. Following exposure to room air, there was no significant difference in 8-isoprostane level in the lung tissue between Antileukinate-treated WT and Tg groups. However, following exposure to 95% O_2_, there was marked elevation of 8-isoprostane in both WT and Tg mice. In WT mice, Antileukinate significantly reduced 8-isoprostane (*p* < 0.05). In Antileukinate-treated Tg group, the 8-isoprostane level was further, significantly decreased compared to the Antileukinate-treated Wt counterpart group after acute hyperoxic exposure (*p* < 0.05). In Tg mice, Antileukinate remarkably decreased the 8-isoprostane level after acute hyperoxic exposure (*p* < 0.05) (Figure 3A).

The ratio of reduced/oxidized glutathione reflects the oxidative status of tissue. Following exposure to air, there was no significant difference in the ratio of reduced/oxidized glutathione in the lung tissue between Antileukinate-treated WT and Tg mice. Following exposure to 95% O_2_, there was marked decrease in the ratio in both WT and Tg mice. In WT mice, Antileukinate significantly increased the ratio (*p* < 0.05). The Antileukinate-treated Tg group showed a further increase in the ratio compared to their Antileukinate-treated WT counterparts after hyperoxic exposure (*p* < 0.05). Notably in Tg mice, Antileukinate revealed significant increase in the ratio after acute hyperoxic exposure (*p* < 0.05) (Figure 3B).

### Antileukinate and/or hEC-SOD overexpression improve alveolar development

**Figure 4:**
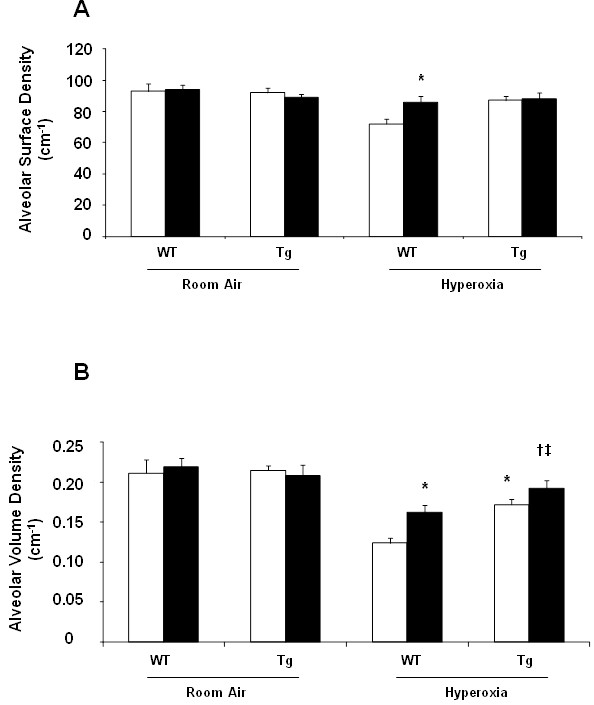
**Alveolar surface density (A) and volume density (B) of WT and Tg mice ± Antileukinate (■ treated, □ non-treated) after 7 day-exposure to air or 95% O**_**2**_**.** Following exposure to 95% O_2_, in WT mice, Antileukinate significantly improved both alveolar surface and volume density. In Tg mice, Antileukinate also significantly improved alveolar volume density. However, there was no difference in alveolar surface density. Tg mice also significantly improved alveolar volume density compared to WT counterpart groups after hyperoxic exposure. Data are mean of five animals per group ± SEM. * *p* < 0.05 when compared to Antileukinate-non-treated WT/H, ^**†**^*p* < 0.05 when compared to Antileukinate-treated WT/H, ^**‡**^*p* < 0.05 when compared to Antileukinate-non-treated Tg/H. (RA: room air, H: hyperoxia).

Antileukinate significantly improved both alveolar surface and volume density (*p* < 0.05) in WT mice exposed to 95% O_2_ for 72 days. However in Tg mice, Antileukinate significantly improved alveolar volume density (*p* < 0.05) alone and there was no significant effect on alveolar surface density. Antileukinate-treated Tg mice also showed significant improvement in alveolar volume density compared to the Antileukinate-treated WT counterpart group following acute hyperoxic exposure (*p* < 0.05). (Figure 4A & B).

### Antileukinate and/or hEC-SOD overexpression reduces alveolar septal thickness inflammatory cellular infiltration after acute hyperoxic lung injury

**Figure 5:**
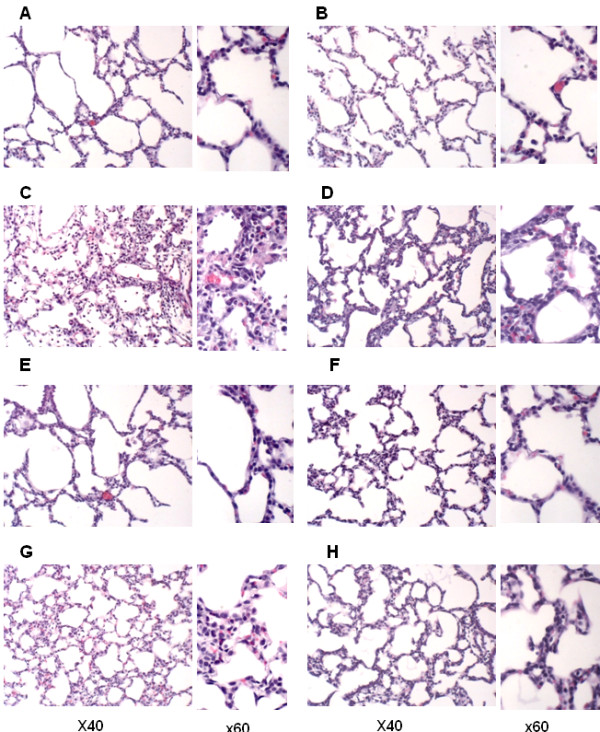
**Representative histologic lung sections from WT in room air (A), Tg in room air (B), WT in hyperoxia (C), Tg in hyperoxia (D), Antileukinate-treated WT in room air (E), Antileukinate-treated Tg in room air (F), Antileukinate-treated WT in hyperoxia (G), Antileukinate-treated Tg in hyperoxia (H).** Hematoxylin–Eosin staining. 95% O_2_ exposed, Antileukinate-non-treated WT (C) revealed thickened alveolar septum and increased cellularity. Other groups showed no evidence of septal thickness inflammatory cell infiltration.

**Figure 6:**
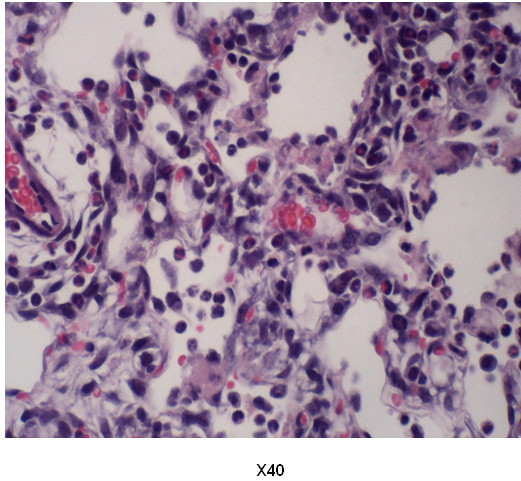
**Histologic lung section from 95% O**_**2**_**exposed, Antileukinate-non-treated WT mice.** Prominent fibrin deposition (arrow) as well as neutrophil (white arrowhead) and macrophage (black arrowhead) infiltration were observed. Hematoxylin–Eosin staining.

Figure 5 represents the histopathology of the lungs from each group. Prominent fibrin deposition as well as inflammatory cell (neutrophil and macrophage) infiltration were observed in the lung sections of hyperoxia Antileukinate-non-treated WT mice (Figures 5C and 6). Other groups demonstrated no evidence of fibrin deposition or inflammatory cell infiltration.

## Discussion

In this study, we tested the hypothesis that the combined strategy of hEC-SOD overexpression and Antileukinate in newborn mice would have a complimentary, protective effect against hyperoxia-induced lung injury. Our data confirmed that when WT and Tg newborn mice were exposed to 95% O_2_, Antileukinate significantly decreased inflammation and oxidative stress, reducing acute inflammation and improving alveolar development.

Studies have indicated the link between hyperoxia and inflammation in the developing animal lung. Hyperoxia-induced lung injury can be considered as a bimodal process resulting from direct oxygen toxicity [11] and from the accumulation of inflammatory cells and mediators within the lungs [12,13]. ROS can damage the lung cells directly, particularly via lipid peroxidation leading to cellular membrane disruption, followed by cellular necrosis. It is known that hyperoxia results in leakage of ROS from the mitochondrial electron transport chain, from the process of hypoxia/reoxygenation, whereby the conversion of xanthine dehydrogenase to oxidase, and from activated neutrophils recruited to the lungs via NAPDH oxidase pathway. In addition, ROS has been shown to be involved in the recruitment of inflammatory cells by inducing prolonged expression of specific neutrophil binding proteins to endothelial cell surfaces in particular Endothelial Leukocyte Adhesion Molecule-1 (ELAM-1) [39] and Granule Membrane Protein-140 (GMP-140) [40]. Recently, the role of ROS as a signaling molecule in activating NF-κB has been illuminated [41-43]. In addition to the known role of EC-SOD as a scavenger of free radicals, its anti-inflammatory role has been shown in an animal model of ischemic limb injury [44]. In this model, EC-SOD overexpression leads to reduce expression of TNFα, IL1α, IL6, MIP2, and MCP-1 cytokine and VCAM, ICAM, P-selectin, and E-selectin adhesion molecule expressions in injured tissues.

Previous studies have shown that hyperoxia exposed hEC-SOD Tg mice typically exhibit around a two- to three-fold increase in total EC-SOD activity over wild-type littermates [17,18]. We have shown previously that augmenting the antioxidant system using hEC-SOD overexpression in newborn mice had a significant impact on hyperoxia-induced lung injury, decreasing the inflammatory response, reducing cell damage and preserving alveolar development [17]. In the present study we have also used direct blockade of the chemokine receptors present on neutrophils. Neutrophils are among the earliest leukocytes to traffic into inflammatory sites and are potent amplifiers of early inflammation, and are recruited to the lung by members of the α-chemokine subfamily [45]. Previously, we have shown also that Antileukinate, a hexapeptide with acetylated amino-terminus and amidated carboxyl terminus (Ac-RRWWCR-NH2), blocks both CXCR1 and 2 and inhibits CXC chemokine–induced neutrophil chemotaxis and activation [22]. We and others have shown that Antileukinate reduces neutrophil associated pulmonary inflammation and injury in a variety of models [28,29,31,34].

In WT mice exposed to 95% O_2_, Antileukinate significantly improved both alveolar surface and volume density. In Tg mice exposed to 95% O_2_, there was a significant synergistic Antileukinate effect on alveolar volume density but no significant effect on alveolar surface density. This could be attributed to the overestimated alveolar development by edema which developed as result of hyperoxia exposure, which will lead to increase alveolar septal volume and hence alveolar volume density or could be due to short term of hyperoxia exposure (7 days). In the Tg hyperoxic group, the effect of hyperoxia is attenuated by EC-SOD overexpression, which will minimize the edematous inflammatory response and its effect on alveolar volume density. The marked significant reduction of both alveolar surface and volume density among hyperoxic Wt groups compared to either Tg alone or treated alone or Tg and treated with Antileukinate hyperoxia groups, highlight the significant protective effect on lung development, which is completely compromised and arrested in hyperoxic injured lung and eventually leads to development of PBD.

McCord et al. have suggested that when neutrophils are activated in the alveolar spaces, the exposure of the endothelium to superoxide increases dramatically as the adherent neutrophils activate their NADPH oxidase [46]. As the neutrophils degranulate, a variety of enzymes is released including myeloperoxidase and a series of proteases. Activated neutrophils induce partial proteolysis of ECSOD-C, resulting in a diminished affinity for the endothelial surface. Antileukinate inhibits both neutrophil chemotaxis and degranulation [27] thus reducing the peroxidase activity and the proteolytic changes. In addition, Liao et al. have shown that neutrophils are a significant source of hydroxyl radical and that blocking neutrophil influx into the lung could substantially reduce downstream products of reactions with hydroxyl radicals, such as 8-isoprostane [26]. Therefore, a further advantage of reducing neutrophil inflammation, in addition to the reduction of interaction of neutrophil components with alveolar cells, is the maintenance of EC-SOD levels and the associated benefits for anti-oxidative stress. Thus blocking neutrophil influx protects the alveolae, at least in part, by preventing hydroxyl radical accumulation and lipid peroxidation.

While Antileukinate is a hexapeptide with a plasma half life of approximately 45 minutes [47], previous studies have demonstrated effective blockade of neutrophil inflammatory responses using at the daily administration of 20–100 *μg*/g [29,31,34]. However, pharmacokinetic studies are needed to determine the optimal dose. In addition, the overall effect of neutrophil blockade on the host immune system is yet to be investigated.

In our current study, we confirmed that overexpression of hEC-SOD or Antileukinate reduced lung inflammation, neutrophil influx and oxidative stress as previous studies have shown. Notably, the level of inflammatory markers and oxidative stress in Antileukinate-treated hEC-SOD transgenic neonatal mice were decreased down to the level of mice on room air. Considering such a complex interplay between ROS, cytokines, and neutrophils, it is not surprising that we can observe dramatic alleviation of lung inflammation when two major pathophysiologic mechanisms of hyperoxic lung injury are targeted.

## Conclusion

The combined strategy of hEC-SOD overexpression and Antileukinate in neonatal mice has an additive protective effect against hyperoxia-induced lung inflammation and reduced oxidative stress in lung tissue after 7 days of hyperoxic exposure. The long term effect of the combined strategy on lung development after hyperoxic exposure still remains to be studied Also the effect of Antileukinate on the immune response and changing the susceptibility of neonates to infection after short and long term treatment approach has to be studied However, we suggest that combination of EC-SOD supplementation and neutrophil blockade can prevent hyperoxia-induced lung injury and may offer an important therapeutic strategy that can protect lung development in premature newborns at risk of developing BPD.

## Competing interests

The authors declare that they have no competing interest.

## Authors’ contributions

JM: carried out the animal experiment and some molecular and genetics studies and participated in data analysis and drafting the manuscript. CC: carried most of the molecular and genetics studies. SN: participated in genetic testing and microscope study. EM: participated in study design and editing the manuscript. MA: design, statistical analysis, data tabulation and editing the manuscript. All Authors read and approved the final manuscript.

Grants: NS-LIJ Pediatrics Department
